# Role of Allergist Advice in Determining Personal Decisions for COVID-19 Vaccination of People With a History of Allergies

**DOI:** 10.7759/cureus.23156

**Published:** 2022-03-14

**Authors:** Polliana Mihaela Leru, Vlad Anton

**Affiliations:** 1 Clinical Department 5, Carol Davila University of Medicine and Pharmacy, Bucharest, ROU; 2 Internal Medicine, Colentina Clinical Hospital/Carol Davila University of Medicine and Pharmacy, Bucharest, ROU; 3 Internal Medicine, Colentina Clinical Hospital, Bucharest, ROU

**Keywords:** history of allergy, covid-19 vaccination, covid-19 pandemic, allergist advise, adverse reactions

## Abstract

Introduction

Vaccination is the most important confirmed tool that can stop the ongoing coronavirus disease 2019 (COVID-19) pandemic, but the vaccination rate remains low in some countries, including Romania, despite consistent and undoubtedly scientific proof of this disease-prevention method. The risk of allergic reactions after COVID-19 vaccines may be a reasonable cause for the antivaccination attitude of people with a history of allergies or who consider themselves at risk for having severe allergic reactions after vaccination.

Objective

Our paper aims to analyze the role of allergist advice in getting people with a history of allergies to trust and accept COVID-19 vaccines and to evaluate the real risk of allergic reactions to these vaccines in this population.

Method

We performed a retrospective study of patients who asked for allergist advice from our hospital before getting one of the COVID-19 vaccines and who received a consultation either through phone call or online or have been admitted for hospital evaluation in 2021.

Results

More than 300 calls and 100 online consultations for COVID-19 vaccination-related allergist advice were done in our center within one year. From the total number of 210 people who were evaluated based on a one-day hospital stay in the Allergology department, 64 patients (30.47%) have been scheduled for evaluation before vaccination because of their past or recent medical history of allergies. Nine patients had documented post-vaccination adverse events, which occurred after the first dose in seven cases, after the second dose in one case, and after the booster in one case. The reactions were mild but one was moderate. No patient was considered to have a contraindication or special precaution for COVID-19 vaccination, and all patients with reactions after the first dose could safely complete the vaccination scheme.

Conclusion

In conclusion, allergist advice and evaluation can significantly influence the decision to vaccinate in patients with a history of allergies.

## Introduction

The first case of coronavirus disease 2019 (COVID-19) in Romania was recorded on February 27, 2020. Until the end of 2021, Romania had recorded 1,808,891 severe acute respiratory syndrome coronavirus 2 (SARS-CoV-2) infections and 58,752 deaths [1].

During the COVID-19 pandemic, Romania went through an extremely difficult medical situation, with the medical system having almost collapsed and a significantly high number of deaths relative to severe COVID-19 forms, mostly during the fourth wave. The fifth wave of COVID-19 due to the Omicron variant is still ongoing, but the decreasing trend of infections enabled national authorities to reduce most of the restrictions imposed on the population while maintaining the indication for continuous vaccination.

COVID-19 vaccination in Romania started on December 27, 2020, shortly after the approval of the first mRNA vaccine from Pfizer BioNTech in the United States [2]. Since the end of 2020, four types of COVID-19 vaccines have been approved in Romania: two mRNA-Pfizer Bio-NTech and Moderna and two recombinant vaccines from Johnson & Johnson and Astra Zeneca.

Despite consistent and undoubtedly scientific data in favor of vaccination, the antivaccine movement has a significant influence in many countries, including Romania, where a rather low vaccination rate has been recorded. It is largely recognized that vaccination accepted by the majority of the population in countries where this was available represented the most important medical tool that can reduce the consequences of SARS-CoV-2 infection in terms of preventing symptomatic and severe disease progression, hospital admissions, and deaths [3]. The vaccination rate in Romania reported on February 2, 2022, was 41.85% from the general population, much lower than in the majority of European countries [4]. This situation could be a potential risk factor for new virus mutations and the occurrence of new COVID-19 waves.

The low COVID-19 vaccination rate in Romania was mostly attributed to some deficiencies of the official public campaign aimed at convincing the population to trust and accept the vaccination, as shown by a recent local study [5]. According to this study, the fear of vaccines’ side effects was mentioned by 21.5% of the respondents to the question concerning reasons to not vaccinate, whereas 40.5% of the respondents showed distrust due to fake news concerning the vaccines.

Consistent data from the literature showed that the risk of allergic reactions associated with vaccines is low and nonallergic reactions, such as fever and local inflammatory reactions, are much more frequent than allergic reactions. Anxiety-related adverse reactions may mimic allergic reactions in some people and can be confused with allergies by patients or nonmedical personnel [6].

Some individuals manifested concern regarding receiving any of the COVID-19 vaccines due to the potential risk of side effects, which might be a reasonable argument against vaccination and has to be consistently addressed by health care professionals [7].

Clinical trials performed with all COVID-19 vaccine types have reported adverse effects, including hypersensitivity reactions, but no fatal cases due to this type of reaction were recorded [8-9].

Reports of severe allergic reactions from the literature raised concern that the new mRNA vaccines involve a higher risk of anaphylaxis in comparison with the adenoviral-vector vaccines, thus explaining the hesitancy of some people to take this type of vaccine.

Our paper aims to analyze the role of allergist advice in determining people with a history of allergies to trust and accept COVID-19 vaccines and to evaluate the real risk of allergic reactions to these vaccines in this population.

## Materials and methods

We performed a retrospective descriptive study of patients who asked for allergist advice from the Allergology department of Colentina Clinical Hospital, Bucharest, before getting one of the COVID-19 vaccines and who received a consultation either through phone call or online or had been admitted for hospital evaluation within 2021.

We recorded the number of people who called and asked for allergist advice before deciding to be vaccinated or for vaccination-related medical problems since the initiation of the COVID-19 vaccination campaign in Romania. The number of phone calls had progressively increased during the first three months, with approximately three to four calls daily.

The second peak of the patients’ calls was recorded in June 2021, one month before the implementation of the European vaccination certificate. More than 300 telephone calls and 100 online consultations for COVID-19 vaccination-related allergist advice were done by the allergist from our center for one year.

Data collection

All data were collected from the files and consultation register of the patients who asked for allergist evaluation related to COVID-19 vaccination. The study group included patients with a past or recent history of allergies who feared postvaccine allergic reactions, both new and known patients from our recordings. We also included patients referred from vaccination centers for evaluation of adverse reactions that occurred after the initial doses of vaccines to assess clinical types, the severity of these reactions, and the safety of completing the vaccination scheme. We recorded the associated diseases based on patients’ history and the clinical forms and severity of reported post-vaccines reactions.

From the beginning of the COVID-19 pandemic, Colentina Clinical Hospital, Bucharest, has become a COVID-19 support unit, with half of its normal capacity, and all non-COVID-19-related hospitalizations being restricted and based on a one-day stay only. The most difficult COVID-19 pandemic periods, i.e., when almost all intensive care units were blocked, were November 2020-January 2021 and September-October 2021. Many medical consultations for non-COVID-19 patients were given online via a platform inaugurated and assisted by the municipality association of medical services and hospitals in Bucharest.

## Results

**Figure 1:**
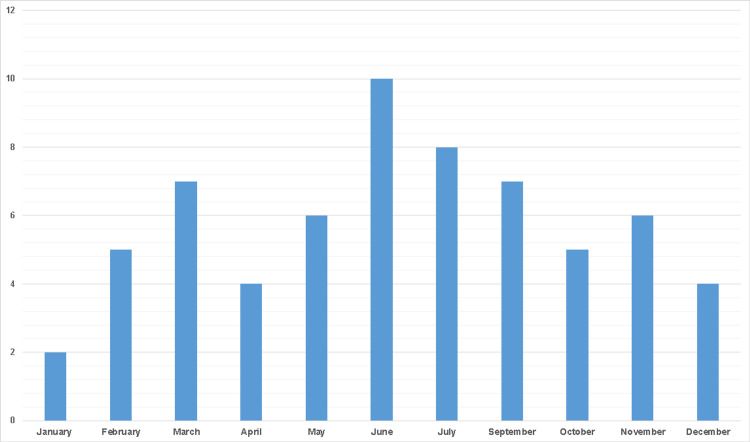
Number of patients addressed for hospital allergist evaluation related to COVID-19 vaccination in 2021

Of the total 210 people who were evaluated based on the one-day hospital stay in the Allergology department, 64 patients (30.47%) were scheduled for allergist evaluation according to patient request and/or indication from referring doctors (Figure 1).

From this patient group, 55 (86%) asked for allergist advice before vaccination owing to their past or recent history of allergies, and 14% were referred from the vaccination centers because of reactions that occurred during the vaccination scheme.

**Figure 2:**
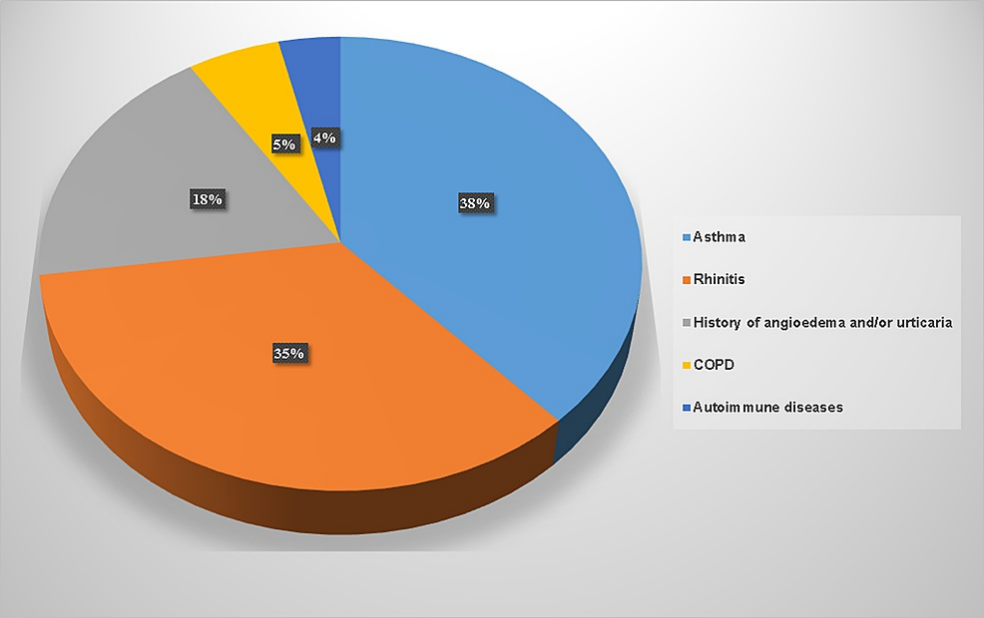
Associated diseases of patients who presented for prevaccination evaluation

The associated diseases of the 55 patients who asked an allergist for evaluation before vaccination were as follows: asthma in 21 patients, rhinitis in 19, history of angioedema and/or urticaria in 10 patients with suspected drug hypersensitivities, chronic obstructive pulmonary disease (COPD) in three, and autoimmune diseases (currently in remission) in two patients (Figure 2).

In the 10 cases who reported a history of angioedema and/or urticaria, all had a past history of mild episodes triggered by medication, either nonsteroidal anti-inflammatory drugs, beta-lactam antibiotics, or angiotensin-converting enzyme inhibitors, which are largely used in adult patients and can be a confounding factor in many angioedema cases [10].

**Figure 3:**
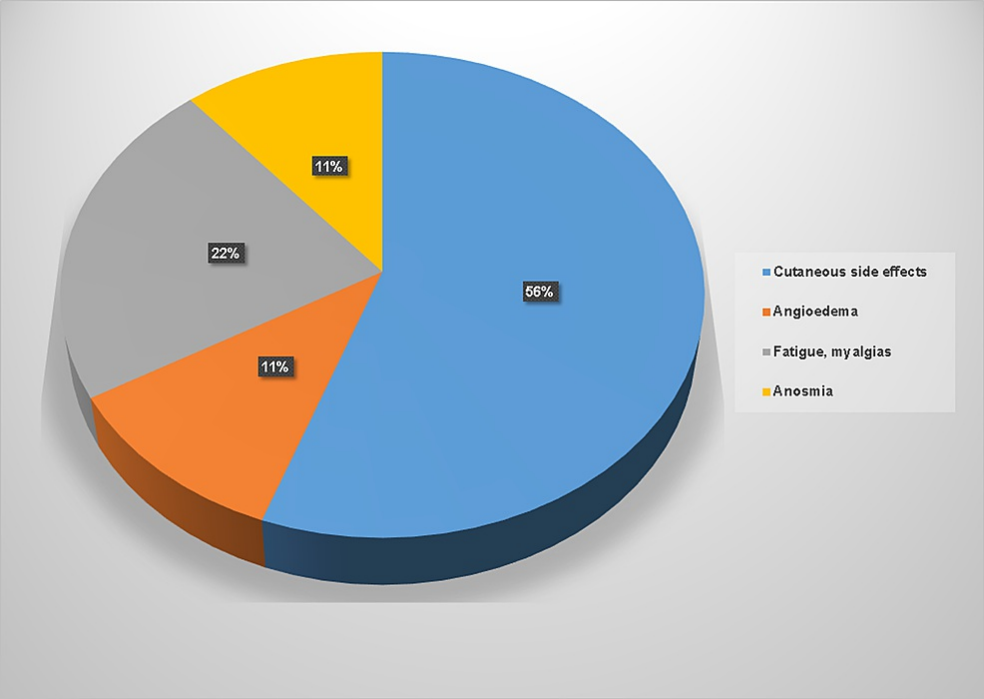
Clinical forms of reported post-vaccination reactions

Regarding the nine cases referred for allergist evaluation of reactions that occurred after vaccination initiation, seven patients received mRNA vaccines and two received adenoidal vector vaccine. We found hypersensitivity cutaneous reactions in five patients and angioedema in one and other side effects related to administration such as myalgia and fatigue in two and persistent anosmia in one patient (Figure 3).

The clinical forms of cutaneous reactions were urticarial rash, fixed erythema, and maculopapular exanthema, with immediate onset in three cases and delayed onset in two cases, and no immediate systemic involvement. These cutaneous reactions were mild and resolved with antihistamines, except one that was considered moderate and needed oral corticosteroids a few weeks. The angioedema case occurred after three weeks of the first dose with a recombinant vaccine, had facial localization (lips), repeated one week after the second dose, and subsided with antihistamines. The one case of persistent anosmia occurred after the second dose of the mRNA vaccine and lasted for almost one year, being resolved with intranasal corticosteroids and antihistamines. All patients who asked for allergist evaluation in our center during 2021 for reasons related to COVID-19 vaccination could receive the recommended complete vaccination scheme.

## Discussion

We noticed that people who asked for allergist advice or evaluation were not against vaccination but had not yet decided because of concerns regarding possible allergic side effects and whose questions were somehow influenced by the allegations of doctors who publicly manifested against vaccination, using the allergy risk as a strong argument. All patients from our study group considered themselves at high risk for anaphylaxis, despite not having any medical confirmation of severe allergies and generally showing a low level of information regarding vaccines’ safe profile, with aspects also mentioned in another local study [11].

A real-life study of patient-reported adverse reactions after COVID-19 vaccination, reflecting the early experience with this vaccination, found that the severe reactions were rare, with a rate of anaphylaxis of 0.3%. The adverse reactions were more frequent for mRNA-1273 vaccines and associated with cofactors such as younger age, female sex, prior COVID-19, Asian race, pregnancy, and marijuana use. Other personal characteristics, such as older age, male sex, Black or African-American race, higher social status, and concomitant asthma or anemia, were associated with lower rates of reported adverse reactions [12].

Case reports of reactions after COVID-19 vaccines have raised concern regarding their safety and risks for allergic or immunocompromised individuals; therefore, particular recommendations were addressed to allergists and immunologists regarding these patients. According to the Guidelines of the Canadian Society of Allergy and Clinical Immunology and the statement of the European Academy of Allergology and Clinical Immunology, allergist evaluation is required for individuals with suspected allergies to a COVID-19 vaccine or any of its components and is not indicated for people with a history of unrelated allergies to foods, drugs, insects’ stings, or environmental allergens [6,13].

The feasibility of allergy testing for the mRNA vaccines is not yet known. These vaccines contain stabilizing substances, such as polyethylene glycol (PEG), which has been identified as potentially allergenic, but the causal relation with reported allergic reactions to vaccines remains unclear [14]. PEG is largely present in a lot of daily use products such as laxatives, cosmetics, foods, and drinks. The Oxford Astra Zeneca vaccines contain polysorbate 80 (P80), which may cross-react with PEG, but the clinical implication remains unclear [15].

The data from epidemiological studies before the COVID-19 pandemic have shown that women and those with a personal history of allergic reactions appeared to have a higher risk for adverse reactions to vaccines, with 85% of vaccine anaphylaxis cases reported in these two categories [16].

There is a clear need for further studies in atopic populations to elucidate mechanisms and assess the risk of allergic reactions and anaphylaxis in these individuals, as well as to assist them with guidelines recommendations regarding vaccine allergy risk [17-18]. The role of allergists appears to be important, considering this task, and is influenced by the number of specialists and the situation of the specialty in each country. Romania has a significantly lower number of allergists and specialized allergy centers, compared to other European countries, as shown by a local study, thus explaining the rather limited availability of allergy evaluation for a large population [19].

Given the continuous global increase in the prevalence of allergies, it is important to note that the anaphylaxis rate in the United States’ general population is estimated to be approximately 2%-5%, most commonly due to drugs, foods, and insect stings [20]. Despite the extreme concern of allergic people regarding the risk of anaphylaxis, the annual incidence of drug-induced anaphylaxis is very low, much lower than the risk of deaths due to COVID-19, which killed more than 5,900,000 people in the world up to now [21].

Based on the comprehensive evaluation performed by experienced allergists, people with various allergic disorders can be informed and secured before taking the vaccines, in terms of the type of the vaccine, possible adverse reactions, available therapeutic approach, and future attitude [22].

The main limitations of our study include the small study group, lack of confirmatory medical documents for past allergies in many patients, unavailability of in vivo allergy tests, and organizational difficulties due to the pandemic.

Despite these limitations, our study has some strengths given that it describes useful and relevant practical aspects related to COVID-19 vaccination from the allergist perspective based on real-life experiences in a supporting hospital from Romania during the second year of the COVID-19 pandemic.

## Conclusions

The results of our study confirmed that anxiety caused by potential severe allergic reactions due to COVID-19 vaccines is an important reason against vaccination in people with a history of allergies, and allergist advice can significantly influence the decision for vaccination in these individuals. Our findings showed a very low rate of allergic reactions after COVID-19 vaccines, the majority being mild, with no real vaccination contraindication in our study group.

Considering the demonstrated safety and effectiveness of the new mRNA COVID-19 vaccines, the balance between their relative risk and benefits has to be mostly evaluated in people with allergies. This involves further efforts of the medical staff and the health system to not only address vaccine hesitancy and advance future vaccination campaigns during the COVID-19 pandemic but also for possible other infectious diseases.
